# Implementing a provider-initiated testing and counselling (PITC) intervention in Cape town, South Africa: a process evaluation using the normalisation process model

**DOI:** 10.1186/1748-5908-8-97

**Published:** 2013-08-26

**Authors:** Natalie Leon, Simon Lewin, Catherine Mathews

**Affiliations:** 1 Health Systems Research Unit (HSRU), Medical Research Council of South Africa (MRC), P.O. Box 19070, Tygerberg, 7505 Cape Town, Republic of South Africa; 2 HSRU, MRC and Global Health Unit, Norwegian Knowledge Centre for the Health Services, Norway, Pilestredet Park 7, 0176 Oslo, Norway; 3 HSRU, MRC and School of Public Health and Family Medicine, University of Cape Town (UCT), Falmouth Building, Anzio Road, Observatory, 7925 Cape Town, Republic of South Africa

**Keywords:** Routine ‘opt-out’ HIV testing, Process evaluation, Normalisation process model, Sexually transmitted infection, Qualitative method

## Abstract

**Background:**

Provider-initiated HIV testing and counselling (PITC) increases HIV testing rates in most settings, but its effect on testing rates varies considerably. This paper reports the findings of a process evaluation of a controlled trial of PITC for people with sexually transmitted infections (STI) attending publicly funded clinics in a low-resource setting in South Africa, where the trial results were lower than anticipated compared to the standard Voluntary Counselling and Testing (VCT) approach.

**Method:**

This longitudinal study used a variety of qualitative methods, including participant observation of project implementation processes, staff focus groups, patient interviews, and observation of clinical practice. Data were content analysed by identifying the main influences shaping the implementation process. The Normalisation Process Model (NPM) was used as a theoretical framework to analyse implementation processes and explain the trial outcomes.

**Results:**

The new PITC intervention became embedded in practice (normalised) during a two-year period (2006 to 2007). Factors that promoted the normalising include strong senior leadership, implementation support, appropriate accountability mechanisms, an intervention design that was responsive to service needs and congruent with professional practice, positive staff and patient perceptions, and a responsive organisational context. Nevertheless, nurses struggled to deploy the intervention efficiently, mainly because of poor sequencing and integration of HIV and STI tasks, a focus on HIV education, tension with a patient-centred communication style, and inadequate training on dealing with the operational challenges. This resulted in longer consultation times, which may account for the low test coverage outcome.

**Conclusion:**

Leadership and implementation support, congruent intervention design, and a responsive organisational context strengthened implementation. Poor compatibility with nurse skills on the level of the clinical consultation may have contributed to limiting the size of the trial outcomes. A close fit between the PITC intervention design and clinical practices, as well as appropriate training, are needed to ensure sustainability of the programme. The use of a theory-driven analysis promotes transferability of the results, and the findings are therefore relevant to the implementation of HIV testing and to the design and evaluation of complex interventions in other settings.

**Trial registration:**

Current controlled trials ISRCTN93692532

## Background

Provider-initiated HIV testing and counselling (PITC) is an approach that has been shown to increase the uptake of HIV testing in a variety of settings [1,2]. However, the impact of PITC interventions ranges widely across settings, and the reasons for this are unclear [1,3]. This study is a process evaluation of a PITC intervention in the context of a controlled trial with the aim of understanding the underlying reasons for the trial outcomes. Process evaluations can benefit from the use of a theoretical model to facilitate a deeper level of explanation and to offer a source of external validity [4,5]. In this study, the Normalisation Process Model (NPM) [6,7] was used as an analytical framework for the analysis of how implementation processes shaped the outcomes of the controlled trial.

### Provider-initiated HIV testing and counselling

PITC, sometimes referred to as ‘routine offer of testing’ or ‘opt-out testing,’ is a streamlined model promoted by the World Health Organisation (WHO) and the Joint United Nations Programme on HIV/AIDS (UNAIDS) to increase the opportunities for diagnosing HIV in health facilities, especially in high prevalence countries [8,9]. The aim of PITC is to decrease barriers to testing in order to increase testing rates and thereby facilitate earlier access to HIV treatment and prevention. Evidence from both low and high income countries indicates that the direct offer of HIV testing by health providers can result in significant improvements in test uptake and that the intervention is acceptable to patients and providers [1,2,10]. Nevertheless, the absolute effect sizes of HIV testing rate range from as low as 5% to as high as 50%, while baseline testing rates can vary from 6% to as high as 75%, depending on setting [1-3,8,11]. The literature offers little explanation for these wide variations in effect size, but suggests that differences in the design of the intervention, the operational context, and the intensity of implementation may play a role [1]. Available studies often do not provide sufficient information on the details of the PITC intervention, making it difficult to compare results across studies.

There are indications that PITC may be effective in Sub-Saharan Africa, but the evidence is limited to a few studies among mainly antenatal and TB patient groups. For example, in Botswana, a significant increase was reported in antenatal patients who knew their HIV status, from 47% to 78% before and after introducing PITC, respectively. In South Africa, studies using a variety of research methodologies indicate that PITC can increase HIV test uptake compared to the standard VCT approach to testing [12-16]. A cluster randomised trial with newly registered TB patients showed an absolute difference in HIV testing uptake at the end of the trial between the intervention and control arms of 13.7% (6.5% in control and 20.2% in intervention clinics, p = 0.009) [16].

In South Africa, with its high prevalence of HIV and low awareness of HIV-positive status [17], the potential value of a PITC approach in expanding HIV testing could be substantial. To maximise the potential benefit of PITC interventions in South Africa, it is important to understand how PITC design and implementation processes shape the outcome of the intervention. It has been noted widely that the key challenge to improving healthcare delivery in South Africa is to improve the implementation and monitoring of effective interventions [18]. Process evaluations alongside controlled trials could improve our understanding of the organisational and technical processes of successful implementation, yet few trials include such evaluations [5,19,20]. A process evaluation of the delivery of PITC could contribute knowledge about when, how and why the PITC intervention worked or failed - evidence that is useful for understanding the outcomes of the PITC intervention in a specific context, for strengthening future implementation strategies, and for developing transferable lessons regarding barriers and facilitators to effective implementation [5,19,21]. This study is the first to report on a process evaluation to investigate the possible underlying reasons for the outcomes of a PITC intervention.

The controlled trial in which this process evaluation is nested was a pragmatic, cluster non-randomised controlled trial, in which 7 clinics were selected to receive the intervention, and 14 clinics served as control clinics. The trial showed that the PITC intervention was successful at increasing test uptake (compared with the standard self-initiated VCT approach) among patients with sexually transmitted infections (STI) in a primary healthcare setting [12]. The study showed an absolute difference in HIV test uptake of 13.8% between a nurse-led PITC intervention and VCT control clinics (56.4% in intervention and 42.6% in control clinics, p = 0.037). Despite the increased test uptake in intervention clinics, the absolute effect size was smaller than the 20% anticipated by health service managers. Table 1 is an extract from the original article reporting the outcomes of the trial by facility [12]. It shows that the proportion of new STI patients who were offered HIV testing in intervention clinics was 76.8%, which was lower than the 100% test coverage expected. However, a significant increase in HIV test uptake was achieved despite the intervention requiring ‘upwards’ task shifting of elements of testing and counselling from lay workers to nurses (as described in more detail below). The process evaluation sought to better understand the reasons underlying these outcomes by examining how the intervention was deployed.

**Table 1:** Trial outcomes per clinic: proportion of new STI patients who were ‘offered HIV testing’, ‘HIV tested,’ ‘not tested’ and ‘declined HIV testing’ in the intervention and control groups

	**New STI patient total**	**Offered HIV testing (%) (Total offered/Total STI)**	**HIV tested (%) (Total tested/Total STI)**	**Not tested for HIV (%) (Total declined/Total STI)**	**Declined HIV testing (%) (Total declined/Total offered)**
**Intervention clinic**					
1	451	363 (80.5)	256 (56.8)	107 (23.7)	107 (29.5)
2	520	400 (76.9)	306 (58.8)	94 (18.1)	94 (23.5)
3	412	346 (84.0)	236 (57.3)	110 (26.7)	110 (31.8)
4	850	572 (67.3)	492 (57.9)	80 (9.4)	80 (14.0)
5	425	338 (79.5)	228 (53.6)	110 (25.9)	110 (32.5)
6	249	215 (86.3)	177 (71.1)	38 (15.3)	38 (17.7)
7	146	92 (63.0)	57 (39.0)	35 (24.0)	35 (38.0)
Total*	**3,053**	**2,326 (76.8)**	**1,752** (**56.4)**	**574 (20.4)**	**574 (26.7)**
**Control clinic**					
8	421	87 (20.7)	86 (20.4)	1 (0.2)	1 (1.1)
9	174	119 (68.4)	105 (60.3)	14 (8.0)	14 (11.8)
10	388	129 (33.2)	120 (30.9)	9 (2.3)	9 (7.0)
11	166	51 (30.7)	47 (28.3)	4 (2.4%)	4 (7.8)
12	593	197 (33.2)	165 (27.8)	32 (5.4)	32 (16.2)
13	789	669 (84.8)	636 (80.6)	33 (4.2)	33 (4.9)
14	837	684 (81.7)	534 (63.8)	150 (17.9)	150 (21.9)
15	626	333 (53.2)	221 (35.3)	112 (17.9)	112 (33.6)
16	320	25 (7.8)	24 (7.5)	1 (0.3)	1 (4.0)
17	373	135 (36.2)	103 (27.6)	32 (8.6)	32 (23.7)
18	164	27 (16.5)	27 (16.5)	0 (0.0)	0 (0)
19	285	211 (74.0)	182 (63.9)	29 (10.2)	29 (13.7)
20	576	449 (78.0)	327 (56.8)	122 (21.2)	122 (27.2)
21	315	290 (92.1)	244 (77.5)	46 (14.6)	46 (15.9)
Total*	**6,027**	**3,406 (50.7)**	**2,821 (42.6)**	**585 (8.10)**	**585 (13.5)**

*Unweighted clinic averages.

### The normalisation process model

The NPM takes a sociological perspective on implementing change in healthcare delivery by focusing attention on those dynamic processes through which complex health interventions are made ‘workable’ and ‘integrated’ to the point of becoming embedded as normal practice, or ‘normalised’ [22,23]. The NPM has been described as a robust conceptual model, its starting point being a systematic re-analysis of qualitative findings from a range of studies of change in clinical practice. Based on this re-analysis, the NPM authors developed cross-cutting analytic constructs and propositions [6,7,22,23]. The NPM has been used to improve understanding of the factors influencing the implementation of complex healthcare provider change interventions in a range of clinical contexts [23-28].

The model identifies four constructs — interactional workability, relational integration, skillset workability, and contextual integration (each with two dimensions) — which influence when, how and why an intervention may become embedded in routine practice or ‘normalised.’ Table 2 provides a summary of the NPM constructs, including their dimensions and propositions about how each construct would contribute to normalisation. The model is described in more detail in the Normalization Process Theory On-line Users’ Manual and Toolkit (http://www.normalizationprocess.org). Since this study was conducted, the model has been reworked into the Normalisation Process Theory (NPT), which includes a number of constructs that were not included in the earlier NPM. Since these changes were made after this work was started, the earlier version of the model was used in this study.

**Table 2:** Constructs and dimensions of the normalisation process model (NPM)

**Constructs**	**Dimensions**
**Interactional workability **The interactional workability construct seeks to examine whether the complex intervention promotes ease and efficiency of interaction between people and practice. The model proposes that the new intervention is more likely to be normalised if the intervention maintains or enhances existing norms and social relations.	**Congruence** Congruence requires shared expectations of normal conduct and purpose of the clinical encounter.
	**Disposal of work** This requires an investigation into the level of agreement about the meaning and consequences of the work and of expectations about its goals.
**Relational integration** The relational workability construct investigates the extent to which the complex intervention can be integrated with existing knowledge, practices and relationships. The model proposes that normalisation is more likely if the intervention maintains or improves accountability and confidence within existing professional networks.	**Accountability** Accountability requires agreement about the validity and expertise of knowledge and role divisions underpinning the work.
	**Confidence**Confidence requires agreement on the credibility and utility of the knowledge and expertise, and the criteria by which it is evaluated.
**Skills-set workability** Skills-set workability is concerned with how the current division of labour is affected by the intervention, the capacity of participants to deploy the required tasks and how the quality of the work is monitored. The model proposes that normalisation is more likely if the intervention has a good fit with an actual or realisable division of labour.	**Allocation** Allocation requires agreement on the formal and informal rules about the assignment of tasks, beliefs about ownership and appraisal of skills, rewards linked to roles and how work is monitored.
	**Performance** Performance involves the ability of the organisation and people to deploy the intervention as planned and includes agreement about the tasks, boundaries, responsibility and autonomy of participants.
**Contextual integration** Contextual integration focuses on how the organisation uses its capacity and resources in the normalisation of the complex intervention. The model proposes that the complex intervention is more likely to be normalised if the organisation is able to be responsive and flexible in executing the work.	**Execution** Refers to the organisational factors influencing practical implementation and monitoring of the intervention. This includes decisions about distributing responsibility, power and resources and linkages to organisational structures.
	**Realisation** Realisation is made possible by agreement about the value of the intervention, policies about procurement, delivery of personnel and equipment and mechanisms for modifying organisational objectives.

(Adapted from May *et. al,* 2007 [23]).

The aim of the study was to investigate how implementation factors may have influenced the outcomes of a controlled trial that aimed to use PITC to increase HIV test uptake amongst STI patients in Cape Town, South Africa. The evaluation (including the controlled trial and process evaluation) was commissioned by the municipal and provincial departments of health in Cape Town. This study describes the deployment of the PITC intervention and how implementation was experienced by various stakeholders. The NPM was used to inform theoretical analysis of the implementation processes for PITC with a view to improving the optimisation of PITC scale-up in the future.

## Methodology

### Study setting

The PITC intervention was delivered in the public sector, primary healthcare services in Cape Town, South Africa, between 2006 and 2007. STI services in this setting are free of charge and are delivered mostly by trained nurse practitioners. At the time of the research, Voluntary Counselling and Testing (VCT) was the standard approach to HIV testing, with patients self-initiating HIV testing or being referred by clinicians. The VCT service was delivered mainly by trained lay health counsellors.

At the start of the intervention in 2006, the total new STI caseload for Cape Town was more than 5,000 patients per month. Although Cape Town has among the lowest HIV prevalence in the country, the prevalence varies dramatically between sub-districts of the city. In 2005, the average HIV prevalence for pregnant women was 12.7% [29], while some of the poorest sub-districts had rates of over 30%, amongst the highest in the country [30]. While HIV testing among pregnant women was high (over 90%) due to the Prevention of Mother to Child Transmission (PMTCT) programme, HIV testing rates amongst STI patients were estimated to be less than 30%.

As part of an effort to expand HIV testing uptake amongst STI patients, the Cape Town health authority initiated a demonstration project, implemented in the context of a controlled trial, to examine the acceptability, feasibility and impact of PITC. The trial aimed to test the effect of the intervention under normal operational and management conditions. The primary outcome of the trial was the HIV testing rate amongst new STI clients. Secondary outcomes were the proportion of STI clients offered HIV testing (irrespective of whether they accepted or not) and the proportion that declined HIV testing, once offered. The findings of the controlled trial were described in detail elsewhere [12]. This process evaluation complements the trial findings; examining the implementation of the PITC intervention could improve our understanding of the trial outcomes.

### The PITC intervention

In this intervention, the STI nurse offered HIV testing as a standard part of STI care for all new STI patients, and the patient had to decline or ‘opt-out’ of this testing. Abbreviated pre-test counselling consisted of informing patients of the common link between HIV and STI (sexual transmission) and recommending that they test for HIV at their STI consultation. If they agreed, the nurse would do a brief test readiness assessment, obtain written informed consent, and perform the HIV rapid test along with other routine blood tests such as those for syphilis.

By using clinical staff to deliver the intervention as an integrated part of the STI consultation, the intervention departed significantly from the standard approach in South Africa, where VCT is predominantly provided by lay counsellors. The shift required a reconfiguration of roles for nurses and lay counsellors. WHO guidelines suggest that a single provider performs both HIV testing and post-test support. However, this PITC intervention stopped short of this, mainly due to concerns about nurses’ time and limited counselling training. To adapt the PITC intervention to the local context and save nurses’ time, nurses were not required to do the post-test counselling. Instead they referred the STI patient to the clinic lay counsellor for the test result and post-test counselling after their STI consultation. It was anticipated that more patients would be tested because the clinical care setting provided the opportunity to offer HIV testing to all patients and because the approach might also increase patients’ willingness to test.

In Table 3, we compare the main components of the PITC approach with the standard VCT approach as it was applied in this setting. The categories for comparison were drawn from a tool developed for PITC in TB settings [31]. These include key implementation elements such as how the service was accessed, who delivered the service, and how counselling and consent procedures were handled.

**Table 3:** Similarities and differences between the VCT and the PITC interventions for STI patients in Cape town

	**Voluntary counselling and testing**	**Provider–initiated testing and counselling for STI patients**
**Patient access**	• Client-initiated: patients come on their own initiative or are medically referred for HIV testing.	• Provider-initiated: patients come to the clinic because they are seeking treatment for STI-related symptoms.
	• Patients anticipate being tested for HIV at their clinic visit.	• The STI nurse offers all STI patients an HIV test, irrespective of their presenting complaint.
**Providers**	• Usually provided by trained lay counsellors.	• Professional healthcare providers (STI nurses) trained to provide PITC.
	• Basic counselling training can be lengthy (10 to 20 days).	• Training is short (2 days) and is focused on how to offer the test and how to get informed consent from patients.
**Primary purpose of the intervention**	• The primary purpose is to promote uptake of HIV testing and to link people to HIV care and prevention services.	• The primary purpose is, similarly, to promote uptake of HIV testing and increase the number of people who know their HIV status.
	• The emphasis is on assessing patient readiness to test, and the counsellor is supposed to remain neutral about the choice (and not to promote taking the HIV test as the preferred option).	• The intervention also aims to integrate HIV testing efficiently into a regular STI consultation, while still respecting the need for patient informed consent.
		• The provider can promote HIV testing as the medically recommended option (rather than remaining neutral about the preferred choice).
**Pre-test encounter**	• Patient-centred counselling techniques focus on promoting an informed decision and include basic HIV information, risk assessment, test-readiness assessment, and risk reduction messages.	• Offer of HIV testing is introduced using regular clinical communication as part of the STI consultation.
	• Written informed consent for testing is obtained.	• This involves a brief explanation of why an HIV test is recommended in the context of an STI consultation, a brief assessment of the patient’s readiness to test for HIV, offering the HIV test and opportunity for the patient to ask questions. Risk assessment and risk reduction are dealt with as part of the regular STI consultation.
	• Can take up to 25 minutes.	• Written informed consent for testing is obtained.
		• Intervention is meant to add maximum 5 to 10 minutes to the STI consultation when efficiently integrated.
**The HIV test**	• Due to limits to their scope of practice, lay counsellors cannot perform the rapid HIV test themselves.	• The nurse does the HIV rapid test along with other blood tests during the STI consultation, which reduces waiting time for patients.
	• The rapid test is performed by a nurse, which may involve some waiting time.	
**Post-test and follow-up care**	• The nurse communicates the result of the rapid HIV test to the lay counsellor.	• The nurse refers the patient to a lay counsellor in the facility, to receive the HIV test result and post-test counselling.
	• The lay counsellor then informs the patient and provides post-test counselling.	• The patient may need to wait for a lay counsellor to be available.
	• The primary focus is on providing emotional support for HIV-positive patients and linking them to care, as well as providing risk reduction messages for HIV-positive and HIV-negative patients.	• The primary focus is similarly on emotional support for HIV-positive patients, but with stronger linkage to HIV care (*e.g*., the nurse does the CD4 blood test on the same day, and the patient is encouraged to attend follow-up sessions with the lay counsellor).
	• Lay counsellors are encouraged to provide up to three follow-up counselling sessions with HIV-positive patients.	• There is less focus on HIV-negative patients.

(Adapted from Table 2 in Bock *et. al*. [31]).

### Study design

The design is a longitudinal process evaluation. We used a range of qualitative data collection methods, as in other process evaluations [5,19]. These methods included staff focus group discussions, in-depth interviews with patients, interviews with management staff, and participant observation of planning sessions, meetings, training, supervision and clinical consultations (Table 4).

**Table 4:** Data collection methods and participants of the process evaluation

**Methods**	**Number of participants**
Focus groups with staff, five months into the trial:	
• HIV Lay counsellors (one group)	8
• STI nurses (one group)	8
Focus groups with staff, 17 months into the trial:	
• HIV lay counsellors (one group)	5
• STI nurses (one group)	7
• Facility managers (one group)	11
• Project leaders (one group)	3
Participant observation during the 2006 to 2007 period. This included reviewing associated documents such as official minutes and researchers’ notes.	The researcher attended multiple meetings (planning, preparation, supervision monitoring, and evaluation)
Participant observation of training: the researcher observed the first round of training for both STI nurses and lay counsellors and selected follow-up training.	10 STI nurses (and their clinical supervisors) and 12 lay counsellors
Interviews with patients.	20
Observation of nurse clinical consultations.	13

### Population and sampling

The study population comprised all the role-players who were involved in the PITC intervention. This included the nursing staff and lay counsellors who implemented it, the project leadership team, clinic facility managers, the TB/HIV/STI clinical supervisors, those responsible for providing HIV counselling training to staff (HIV trainers), and patients who received the PITC intervention.

All the STI nurses and at least one lay counsellor from each participating clinic formed the sample for the focus groups. Patients were recruited for interviews using convenience sampling, either after their HIV testing experience or upon their return to the clinic for follow-up services. Nurses from the four largest participating intervention clinics were also observed (clinics number 2, 3, 4 and 5 in Table 1). These clinics were purposely chosen as their high workloads were representative of challenges faced in public sector clinics when introducing new interventions. Systematic sampling was used to select clinical consultations for observation; the researcher attempted to observe the first one or two consultations after arrival at the clinic on each data collection day.

### Data collection

Much of the data on the implementation process of the intervention was derived from the lead researcher’s participant observer role. The observation period stretched over a period of approximately two years and included observations of meetings during the planning and training phases and of implementation activities during the 21 months in which the intervention was deployed. The researcher was able to accompany the project manager on supervisory visits and to sit in on monitoring and evaluation meetings.

Table 4 details the data sources. Altogether, 6 focus groups were conducted with 42 participants. Two focus groups each were conducted with nurses and lay counsellors, one towards the beginning and one towards the end of the implementation period (at 5 months and 17 months post implementation, respectively). At the end of the intervention, one focus group was conducted with facility managers and one with the project leadership (senior manager, project manager, and the new senior manager). These focus groups explored the experiences and perceptions of the participants and their views about the strengths and limitations of the implementation process for PITC.

Twenty individual patient interviews were conducted to examine their experiences of the intervention. We sampled patients who had accepted and who had declined the HIV testing offer. The interviews explored their perceptions of the intervention and the reasons for their test decision-making. To examine how nurses were deploying the intervention during consultations, the researcher examined 13 clinical consultations, through direct observation or digital recording of the consultations. This involved studying five different STI nurses in 4 out of the 7 intervention facilities. The process evaluation draws on, but does not report in-depth, on the data from the clinical consultations and patient interviews [32]. These data will be the focus of subsequent papers.

### Data analysis

All patient interviews and clinical consultations were audio-recorded, transcribed and where required, translated into English from isiXhoso (the local language used by most patients and staff in these clinics). Thematic content analysis was used to analyse the implementation process [33]. Transcripts of focus groups, patient interviews, and clinical consultations were read and re-read to identify the common responses in relation to the key areas, and these, in turn, contributed to development of the main themes. In the first stage of the analysis, a general descriptive framework of factors identified as key for successful implementation (described in Grol and Wensing [21]) was used to identify key barriers and enablers to the intervention. This helped to summarise, organise and categorise the data (see Table 5), and the NPM was used as a theoretical framework in the subsequent phase of the analysis. Explanatory themes were grouped and analysed under the headings of the four NPM constructs, as illustrated in Table 6. The theoretical constructs provided an organising framework for analysing and interpreting the findings. It also provided a coherent and systematic language for describing the factors and the relationships between them, thereby contributing to the transferability of the findings [34].

**Table 5:** Stages of the PITC implementation process and key factors shaping the deployment of the intervention during each stage

**Stages of the PITC implementation process**	**Key factors shaping the deployment of the PITC intervention**
**Stage 1 (August to October 2005)**	
**Project initiation and preparation**	**Credibility, ownership and framing the project by top management**
• The HIV manager of the municipal health department identified a gap in HIV testing uptake for STI patients. She rallied managerial colleagues to motivate for the implementing of the PITC intervention in a demonstration project.	• The project was initiated by the health department itself and not by an external research organisation.
	• The person who initiated the project was a senior manager (the HIV/TB manager) with a track record of achieving quality improvements in the TB/HIV and STI programmes.
• The project aim was to assess the feasibility, effectiveness and efficiency of the PITC intervention in an operational setting.	• The PITC intervention was based on recommendations made in the WHO draft guideline for PITC in 2006.
	• The PITC intervention was promoted as being necessary to enhance comprehensive STI care and in response to real human resource constraints.
**Governance accountability structure established**	**Governance, leadership and accountability mechanisms were in place**
• A project governance structure, the Project Steering Committee (PSC), provided oversight of the planning, implementation, monitoring and evaluation of the PITC project.	• The PSC provided a structured governance mechanism for the participation, collaboration and accountability of relevant stakeholders, including managing conflicting views.
	• The PSC was chaired by HIV manager, who was the initiator, project leader, and who acted as the champion for the project.
• The PSC comprised frontline clinical staff (nurses and lay counsellors), clinical supervisors, clinic management, HIV counselling trainers, project management and the project leader.	• The PSC met at quarterly intervals and provided meetings of the PSC, provided opportunity for continuous monitoring and evaluation, regular feedback and motivation.
**Stage 2 (October 2005 to March 2006)**	
**Planning and project management mechanisms**	**Detailed planning, flexibility and management support provided**
• There was a lengthy planning process spanning nearly nine months prior to implementation as well a detailed operational planning.	• Planning was a ‘start and stop’ process due to disagreements among stakeholders about the acceptability and relevance of the PITC intervention.
	• Facility managers and frontline staff had the flexibility to re-design patient flows in their clinics that would best accommodate the integration of the HIV offer into the STI consultation.
	• Staff requested the support of a project manager to ensure effective implementation and monitoring and evaluation. Management responded positively (contextual integration).
• To strengthen the project management, a project manager was allocated on a part-time basis to be responsible for coordinating the operational level implementation and monitoring.	
**Stage 3 (January to April 2006)**	
**Design of the PITC intervention**	**Local adaptation, contestation and compromise enhancing the acceptability and feasibility of the PITC intervention**
• The WHO version of the PITC intervention had to be adapted on several levels to fit with the local requirements.	• The adaptation of the PITC in intervention was done on several levels geared towards improving the feasibility and acceptability of the intervention. (Upwards task shifting and task sharing).
• The intervention involved re-allocation of roles between clinical staff and lay health workers.	
	• There were several areas of disagreement amongst stakeholders in the PSC regarding the design of the intervention, task re-allocation, and training. The clinical guideline was lengthened to accommodate concerns among HIV trainers regarding ethical implementation of PITC.
• A clinical guideline for nurses was developed to guide their practice in the consultation.	• The above conflicts threatened the feasibility of implementing the project.
	• The conflict resolution and leadership skills shown by the project leader were largely responsible for the successful resolution of conflicts: using compromise and executive decision-making.
**Training**	**Training coverage and feasibility**
• The frontline STI nurses and lay counsellors, as well as a few clinical supervisors, were trained on the PITC intervention by trainers from an HIV counselling training unit within the health department.	• Training was well attended not only by the STI nurses responsible for implementation, but also by their immediate clinical supervisors (district HIV/TB coordinators).
	• Training course for nurses was 2.5 days (reduced from 5 days initially suggested by trainers).
	• Lay counsellors received training to provide more in-depth post-test counselling over two to three counselling sessions per patient.
**Stage 4 (April 2006 to December 2007)**	
**• Health facility-based implementation and monitoring and evaluation**	**Early and continuous monitoring, feedback and support provided**
• Implementation started April 2006 in seven health facilities	• Monitoring and evaluation mechanisms were in place from the start and were continuous throughout the duration of the intervention.
• The monitoring and evaluation systems were planned from the start, including the outcome indicators and the data sources.	• Project support was provided through quarterly ‘cluster’ monitoring meetings that were conducted by staff from two or three clinics at time.
• A quarterly review meeting of the PSC was conducted where all facilities were provided with feedback on progress.	• In cluster meetings and in quarterly PSC meetings, nurses and facility managers reviewed progress (based on routine health information), shared best practices, and addressed practical problems (*e.g*., ensuring supplies of test-kits, testing registers and clinical guideline sheets).
**Evaluation of staff and patient experiences**	**Evaluation of multiple dimensions provided information on perspectives and experiences of important stakeholders.**
• Evaluations of patient and staff perspective and experience were conducted through various qualitative research methods.	• Patient satisfaction surveys and patient exit interviews were done midway to explore the acceptability of the PITC intervention.
	• Evaluation of staff perspective was conducted via focus groups, to explore the acceptability of and the barriers and facilitators to implementation.
	• STI clinical consultations of nurses were observed to examine the delivery of the intervention in terms of efficiency of integration and the quality of informed consent processes.

**Table 6:** Overview, using NPM constructs, of promoting factors and potential threats to normalisation of the PITC intervention

	**Promoting factors**	**Potential threats and how these were addressed**
**Interactional workability**		
**Congruence**	• The design of the intervention was congruent with both operational needs (too few lay counsellors) and STI clinical practice.	• It is difficult to justify upwards task shifting (from lay counsellors to nurses) in a low resource setting, so the PITC intervention was adapted to minimise the increased workload on nurses.
**Disposal**	• Nurses saw this as an opportunity to enhance the standard of STI care.	• It was critical that nurses accepted the paradigm shift toward normalising HIV testing. The project champion achieved this by convincing nurses of the benefits and the feasibility of a shift in practice towards integrating HIV testing.
**Relational integration**		
**Accountability**	• There was a governance structure responsible for stakeholder involvement, planning and oversight.	• The downside of this accountability structure and consultative planning was that it resulted in a long, protracted and fragmented planning phase that delayed the implementation date.
	• Leadership by senior management promoted ownership.	• There was a range of disagreements among stakeholders. The conflicts threatened the viability of the project. Conflict resolution involved a compromise: to extend the clinical guideline and shorten the training. Removal of these stumbling blocks was largely due to the conflict resolution skills of the project champion and because she had the seniority to make executive decisions.
	• The project was provided with a dedicated project manager to support implementation and monitoring.	
**Confidence**	• Nurses were convinced of the utility and feasibility of new intervention, even though they were concerned about the additional workload.	• Lay counsellors and trainers were less confident about the new PITC intervention (see ‘Skill-set workability’ below).
**Skills-set workability**		
**Allocation**	• The new tasks for nurses were in line with standard STI practice.	• Lay counsellors were concerned about their reduced role in pre-test counselling and their job security. This concern was allayed because they were allocated an increased role in the post-test counselling of HIV-positive patients.
	• All the parties agreed that the intervention required training. • Training was well attended by nurses and their clinical supervisors.	
		• Lay counsellors and HIV trainers were concerned about the acceptability, ethics and feasibility of PITC intervention. They agreed to support the intervention only when the clinical guideline was adapted to focus more on assessing the patient’s test readiness. The adapted clinical guideline meant nurses had to include more questions and tasks, making the intervention longer and more difficult to integrate efficiently into the STI consultation.
		• Training focused on counselling skills and did not address the operational challenges of integrating the new HIV tasks within the clinical consultation.
**Performance**	• The HIV offer was delivered to the majority of new STI patients in an ethical manner.	• The HIV test was not offered to all new STI patients as intended, reducing the size of the impact.
	• Levels of confidence and efficiency of delivering the intervention varied with gaps in clinical communication skills evident.	• There was variation in the how efficiently individual nurses delivered the intervention and in how long it took. Although positive about the intervention, nurses remained concerned about the added time.
	• Nurses persisted with intervention despite the challenges around how to balance the new tasks.	
**Contextual integration**		
**Execution**	• Receptive environment for a paradigm shift toward normalising HIV testing.	• The feasibility of this intervention depended on management identifying extra capacity in terms of nurse time, which may be difficult to do in many similar PHC settings.
	• Operational conditions promoted shift toward expanding HIV testing.	
		• Not all the variation in the HIV testing outcomes across intervention clinics could be fully explained, and some may be due to organisational factors.
**Realisation**	• Organisational leadership and accountability in place.	• Dedicated project management support may not be a sustainable component to up-scaling.
	• Responsive resourcing of the intervention through dedicated project management.	• No cost-effectiveness evaluation was conducted.
	• Facility managers reinforced line management accountability.	

Multiple sources of data, such as from staff focus groups as well as from observation of clinical consultations and from patient interviews, allowed for data triangulation. In addition, the project leader and project manager were asked to comment on a draft report, which improved the credibility of the findings.

### Ethics

Ethical approval for the research was obtained from the University of Cape Town Research Ethics Committee. The local and provincial health authorities of Cape Town gave permission for the research and the controlled trial was registered (Trial registration number: ISRCTN93692532). Written consent was required from staff who participated in focus groups and observation of clinical practice. Patients provided written consent for participation in patient interviews and verbal consent for their consultation to be observed.

## Results and discussion

Drawing on a framework developed by Grol and Wensing [21], Table 5 shows the first level of analysis, which describes the implementation stages. For each stage of the PITC implementation process, we identified key factors that that may have shaped successful deployment of the PITC intervention. Building on this initial analysis, we used the theoretical constructs of the NPM to discuss the dynamic interaction of these factors and how they may have influenced the normalisation of the new PITC intervention and the trial outcomes. These findings are summarised in Table 6.

### Interactional workability: to what extent did the new PITC intervention maintain and/or enhance existing norms?

The new PITC intervention was initiated by the senior manager responsible for the development of integrated service delivery for HIV, STI and TB services. This manager lobbied top management to support the introduction of the PITC intervention and to use it to demonstrate feasibility and influence a policy shift. She convened all the stakeholders and took the leadership role in driving the planning and implementation process. Prior to initiating the PITC project, the senior manager outlined her vision for change in HIV testing services [35]. She called for a ‘paradigm shift’ away from treating HIV as an exceptional disease and spoke of the need to increase opportunities for HIV testing in the clinic setting: ‘*Treating HIV testing in this exceptional way contributes to the secrecy and stigma associated with HIV, discourages HIV testing and limits access to the credible treatment options available*’ [35]. She elaborated on how the new PITC intervention could dovetail with a range of existing clinical services, including STI treatment: ‘*Within a medical context this translates to more routine, service provider-initiated HIV testing as part of the standard of care provided in a range of services including the management of sexually transmitted infections, antenatal, reproductive health and TB services, amongst others*’ [35].

The strong leadership role of the senior manager could be described as that of a project ‘champion’ (a term used in literature on diffusion of innovations [36]), and the term ‘champion’ will henceforth be used to refer to the senior manager. The data extracts above show how the project champion framed the new intervention as both responding to a service need (to increase HIV testing opportunities) and to the need to routinise HIV testing within standard clinical practice. Based on routine operational data, she demonstrated that the numbers of lay counsellors available were not sufficient to expand routine HIV testing to all STI patients and that this gap could be filled through minor changes to STI nurse practice.

In effect, the champion framed the new PITC intervention as not only a requirement, but also as a feasible and desirable way of improving HIV testing service delivery. The framing of PITC as both an opportunity to address a service gap and an opportunity to improve the standard of STI care may have strengthened the willingness of nurses to consider its implementation. Nurses were acutely aware that the current VCT system did not provide optimal access and resulted in missed opportunities for HIV testing for STI patients. Appeals to improving clinical care resonate with professional norms about providing quality services and, as such, are likely to have strengthened the acceptability and the interactional workability of the new intervention. A STI nurse explained her experience of the gap in HIV testing for STI patients: *‘When I used to refer them [STI patients] for VCT, they always say: “Oh, how long will I have to wait? Do I have to see a counsellor?”’* The nurse elaborated: *‘That was always an issue. They would say: “I am willing to take the test, but if I have to go to the lay counsellor, I’m not going to test”… it's too time consuming for them.’*

Although the new PITC intervention initially required a paradigm and practice shift, the project champion was able to frame this as not only congruent with, but as enhancing STI clinical practice. This congruence with professional roles may have contributed to nurses internalising the need for change more easily, thus improving the chance of the new intervention becoming embedded in routine practice.

Nevertheless, not all of the stakeholders were supportive of the new intervention, and some disagreements posed a threat to the normalisation of the intervention. During the planning of and training for the new intervention, disagreements emerged between stakeholders about the utility and ethics of PITC, as well as about the changes in role-divisions. STI nurses were now asked to expand their set of tasks to include routinely offering an HIV test, obtaining informed consent, doing a ‘test readiness’ assessment, and performing the finger prick blood test for HIV. Except for the blood test, all of these tasks were previously performed by lay counsellors. Lay counsellors initially felt that the changes in role divisions threatened their job security, but were reassured by their continued and extended role in relation to post-test counselling. However, two other concerns, mainly from HIV trainers, were less easily resolved. One was an ethical concern about reducing pre-test counselling requirements (an issue raised in broader debates on the ethics of PITC [37,38]). The other related concern was about whether nurses had the appropriate skillset to offer HIV testing and, in particular, whether nurses would be able to facilitate ‘true’ patient informed consent. An HIV trainer explains: *‘Already overburdened staff may jeopardise the objectives of this new model because they have a lot of other things to do and the patient may not feel they can refuse.’*

As a compromise, the project leadership agreed to expand the clinical guideline that was developed to support nurses. The guideline was expanded to have a greater focus on assessing test readiness and patient informed consent. In practice, this meant that the nurse was required to ask more questions, provide more HIV information and engage more with the patient, which resulted in a guideline that would take more time than in the original intervention design. As will be discussed later, the longer clinical guideline may have contributed to reducing the feasibility of the intervention on the level of the clinical consultation (see ‘Skillset workability’).

Nevertheless, nurses, as the main implementers, were willing to proceed despite their own concerns about how the new tasks might add to their workload. Nurses in a focus group at the start of the intervention were generally positive toward the change. A STI nurse commented: *‘There are limitations, like the time constraints, but we should go ahead. We should integrate routine screening to all parts of medical care.’*

### Relational integration: to what extent was there accountability for the deployment of, and confidence in, the utility of the new PITC intervention?

A project governance structure was created at the start of the project, in the form of a Project Steering Committee (PSC). The Committee was chaired by the project champion. The PSC had representation from a range of stakeholders, including frontline nursing staff and lay counsellors, their clinical supervisors, HIV counselling trainers, and facility management. The committee was responsible for the planning, coordination, monitoring and evaluation of the project. It remained active throughout the lifespan of the project and provided both ongoing motivation and continuity.

The PSC seemed to have played a central role in strengthening the implementation process. Senior management leadership of the PSC meant there was high-level responsibility for implementation, which would have contributed to its legitimacy. This and the consultative planning process would have inspired confidence in the intervention among participants. Some of the ways in which the PSC supported implementation include raising awareness and keeping participants motivated throughout; identifying and resolving conflicts; strengthening ownership of the intervention; building trust among participants; as well as providing a mechanism for continuous monitoring.

Normalisation was also supported by the appointment of a dedicated project manager, who provided logistical support for implementation and for monitoring and evaluation. Project support was delivered via site visits at clinics as well as through quarterly ‘cluster’ meetings with smaller groups of staff from two or three clinics at a time. These cluster meetings were considered a key mechanism for promoting accountability and confidence amongst staff, as the project manager explained: ‘*The cluster meetings were the “engine” of the implementation process. It was used to share best practice and staff made it their responsibility to bring information to the meeting. They reviewed their monthly and quarterly statistics, they did problem solving and it was considered a team approach.’*

While implementation leadership and support would have strengthened staff confidence, nurses may also have been motivated by their own sense of the utility of the new intervention. When asked to reflect on the purpose of the new intervention, a nurse pointed to the opportunity to improve healthcare delivery: ‘*This is to be able to treat STI fully. It will ensure that clients are offered all services, even HIV testing… we want to offer complete care and treatment’*. Another nurse commented on the feasibility of the PITC intervention, highlighting the close fit with the routine clinical care that she delivered: ‘*With me, even though there wasn’t this project, whenever I was treating an STI, I always talked about HIV. So it’s not a new thing. The only opportunity I have now is that I have to do the HIV test’.* These positive perceptions of the new intervention were commonly expressed in focus groups with nurses at the beginning, during implementation, and at the end of the project – an indication that they believed in the utility of the intervention and saw it as congruent with their existing knowledge, practices and relationships. This suggests that the intervention had strong relational integration, a factor that would have supported normalisation.

### Skillset workability: to what extent did the required skillset fit with existing skillsets and the division of labour?

When asked to reflect on their capacity for implementing these new tasks, nurses consistently pointed to the congruence with standard STI care. As one nurse explained:

‘I think that we always did talk about HIV. People know it’s a virus, they know it has to do with sexual transmission. But I think we spend more time now pointing to the benefit to the patient of testing. Previously we would refer them to VCT and because we weren’t really involved with the testing, we didn’t go into depth such as discussing issues like, “How this is going to benefit you. What services are available for an HIV-positive patient, whether they know how they can prolong their life.”’

While nurses perceived congruence with standard care and did not identify specific training gaps, in practice there were challenges with executing tasks efficiently. Observation of their clinical practice indicated that although they were able to perform all the new tasks (such as offering the HIV test, getting written informed consent, and performing the rapid test) they struggled to do so in an integrated manner. Most commonly, there was an awkward sequencing of tasks in terms of timing and flow, leading to a fragmented approach and a lack of smooth integration with STI tasks. For instance, nurses would sometimes separate their assessment of the patients’ risk for STI from risk assessment for HIV; and health education for prevention of STI and HIV was often separated into different conversations. One of the tasks that took up a good portion of nurses’ time was sharing basic HIV awareness information with the patient, which they did in fairly formulaic fashion, even when this did not seem to be required. As a result of these challenges, the additional HIV-related tasks added more time to the consultation than nurses and the project leadership had planned for.

When nurses were asked about the challenges posed by the intervention, they indicated that the added time was the main obstacle. The main reason for this, they said, was that they were required to convey too much information: ‘*We have to do too much talking and writing’.* Related to this was the greater interaction with patients as a consequence of introducing HIV into the STI consultation: ‘*We have a lot of information to discuss with them, and the clients are also asking a lot of questions’.* Another nurse linked the change in the interaction with patients to an increase in consultation time: ‘*I think it boils down to the fact that we are actually spending more time with each client… As you’re talking you win their trust, and they are perhaps giving you information that they wouldn’t have given before’.*

On average, the new tasks added 7 to 10 minutes more to the STI consultation time (ranging from 3 minutes in one case to as much as 20 minutes of extra time in another), which would have made it difficult to initiate the HIV test offer with every new STI patient. This may explain the trial finding that the proportion of STI patients offered HIV testing (also referred to as the HIV test coverage) was 76.8% for the intervention arm – less than the 100% coverage anticipated (see Table 1[12]). Reduced HIV test coverage would, in turn, have reduced the potential number of people who could accept HIV testing, thus also limiting the size of the main trial outcome (the number of new STI patients who accepted HIV testing). The proportion of new STI patients who were tested in the intervention clinics was just over half, at 56.4% (see Table 1). This points to the importance of achieving a high level of skillset workability for effective implementation, a challenge that we argue was only partly achieved with the PITC intervention.

To illustrate the challenge of skillset workability further, we provide extracts from two clinical consultations to show the range of ways in which nurses approached the challenge of integrating the new tasks. The level of efficiency of deployment varied among nurses observed, most often resulting in long extensions to consultation times. In one clinic where two nurses were observed, one of the nurses seemed more efficient about integrating the HIV test offer compared with other nurses. She achieved this by introducing the HIV test offer early in the STI consultation and by explaining briefly why HIV testing was a good idea when presenting with an STI complaint. In the extract below, the nurse introduced the topic of HIV testing shortly after asking the patient for the reason for their visit: *‘Have you ever tested (for HIV)? Have you ever heard of HIV?’* To this, the patient answered*, ‘Yes’.* The nurse continued with a brief explanation of why an HIV test would be an appropriate medical option to consider: *‘HIV is the same as other diseases that are transferred though sex,’* and further, that *‘you could get HIV because you are not using a condom*’. After a few more lines of explanation about the importance of HIV testing, the nurse asked the patient directly to consider an HIV test: *‘How do you feel about testing for HIV?’* To this, the patient answered*, ‘I could test, there is no problem’.* As the patient agreed to test early in the session, the nurse then completed the written consent requirements and was able to integrate the technical task of performing the rapid HIV test more efficiently. For example, during the time required for the HIV test result to be processed (10 to 15 minutes), the nurse continued with the STI examination and other STI tasks.

In an example of less efficient practice, the nurse in the extract below also introduced the topic of HIV early on. However, she did not immediately link it to the offer of an HIV test, thereby missing an opportunity for smooth integration. Instead, she focussed on dispensing HIV information in an apparent attempt to ensure that the patient was ‘fully’ informed: ‘*Right then. Here it is important for us to let you know more about what made you come here. The STI means sicknesses affecting your private parts, an example being gonorrhea and others, okay? HIV is also included, like you hear around people talking about HIV and AIDS nowadays’.* The patient said, *‘Yes,’* to which the nurse continued, *‘Okay, you must understand that these sicknesses like HIV and STI and TB are all related. They are like cousins, if you understand what I mean’.* The nurse then proceeded with a lengthy explanation about HIV transmission and how the virus works in the body. This was done in a way that was at times circuitous. She made the offer of HIV testing only after much time was spent on providing general HIV health education: *‘Okay. So now that I have explained everything to you, would you be interested to be tested today?’* After the patient agreed to test for HIV, the nurse struggled to efficiently integrate the tasks associated with obtaining written patient consent and assessing the patient’s test readiness. The additional time spent on HIV education occupied a considerable proportion of the consultation time, and this, together with the lack of smooth sequencing with STI tasks, resulted in a lengthy extension to the consultation.

As illustrated above, in the context of clinics with high STI patient loads, the interrelated challenges of integrating new tasks and keeping consultations to a manageable length may have limited the nurses’ ability to offer HIV testing more widely. The long length of the clinical guideline for PITC appears to have reduced the skillset workability of the new intervention, making it more difficult to execute it efficiently.

Another factor contributing to reduced skillset workability was the training. There was disagreement between the project leadership and the HIV trainers about the duration and content of the training for the STI nurses. It was evident from participant observation that training focussed on teaching nurses counselling skills so that they could assess patient readiness and facilitate ‘true’ informed consent. Most of the time was spent on trying to rapidly teach nurses to use patient-centred counselling techniques and on how to provide health education to ensure that the patient was fully informed. In contrast, there was little to no guidance in the training on managing the operational challenge of efficiently integrating the new tasks into a standard STI consultation.

In sum, it would seem that, on a practical level, the new intervention challenged the conventional provider-patient communication used in the STI consultation. For the most part, nurses used a more provider-centred communication style in which they took the authoritative role of providing knowledge and advice. However, the tasks linked to assessing patient readiness for testing and obtaining patient informed consent required more patient-centred forms of communication and did not fit easily with task-orientated nature of the STI consultation. The tension between these two styles challenged the clinical communication skills of nurses and limited their ability to integrate the tasks efficiently. Poor compatibility between the new requirements, existing skills and the clinical context of the STI consultation points to a problem of reduced skillset workability on an operational level. Within a busy PHC setting, with limited clinical personnel, this could be the underlying reason why nurses did not consistently offer the HIV test to all patients, thus limiting the coverage and impacting on the main trial outcome: the proportion of STI patients who tested for HIV.

Despite these challenges with managing their time and extending the intervention to all patients, nurses continued to deliver the intervention for the full duration of the demonstration project. According to the project leadership (champion and project manager), nurses had internalised the paradigm shift towards providing HIV testing as part of routine medical care (rather than as a specialised service), and this is what kept them motivated to continue. Also, as nurses started to offer HIV testing, their increased exposure to HIV issues may have further reinforced their willingness to continue with this change in their practice. The project champion explains: *‘I think it is due to HIV becoming more of an important issue in the sites. It raised staff awareness. When staff deals with HIV themselves, instead of others dealing with it, it makes them more aware of the urgency of testing and this awareness increased staff willingness to deal with HIV testing’.* Although there were important gaps in skillset workability, there was sufficient congruence for this to also be considered a promoting factor. The project manager argued that, despite the increased workload, nurses may have considered the intervention to be an appropriate change in the division of labour and an opportunity to enhance professional practice. She explained: 

‘*I think staff felt empowered to deal with HIV, which was a “no-go” area for them previously, and the responsibility of a different category of staff [lay counsellors]. Being allowed to offer HIV testing empowered them and made them feel they could deal holistically with patients’.*

Nurses also pointed out that the positive reactions of patients provided them with further motivation to continue with the intervention. A patient survey conducted in the early stages of implementation indicated that patients were positive about the PITC intervention [39]. In patient interviews, they expressed appreciation for the opportunity to test for HIV within the STI consultation, finding it more convenient than self-initiated VCT. They expressed appreciation for the nurses’ advice, found it helpful to be reminded of their risk for HIV, and thought the STI consultation was a good opportunity for HIV testing. They reported that they did not experience any sense of coercion in relation to their test decision-making [32].

### Contextual integration: to what extent was the organisational context able to support the new PITC intervention?

The WHO draft PITC guidelines of 2006 signalled a global shift toward the normalising of HIV testing. At the time, the South African National Health Department (NDOH) was beginning to embrace the idea of expanding test uptake in medical settings through revisions to their HIV testing policy [40]. It could be argued that these initiatives provided a receptive international and local context for introducing and executing the PITC intervention in South Africa.

On an organisational level, several other factors may also have contributed toward embedding the new intervention into routine practice, such as appropriate resourcing of the intervention. No extra clinical staff (STI nurses) were made available as it was intended that the intervention be tested in a realistic operational setting where clinical resources were limited. Nevertheless, the dedicated project management support, even if part-time, was an important resource to ensure successful implementation.

Of interest is that the clinic with the smallest STI patient load (Clinic 7 in Table 1) was also the one with the lowest test coverage. Although the study did not investigate nurse practice in this facility, information shared in the cluster meetings indicates that the low test coverage may be due to the absence of a dedicated STI nurse role. The small size of the clinic meant that the one or two nurses in attendance had to treat all types of medical conditions, not only STI patients. This way of organising the service, with a mixed role in a busy setting, may have been an added barrier to nurses offering HIV testing more widely to their STI patients.

Finally, normalisation may also have been strengthened by the role of clinic facility managers as these line managers are responsible for key performance outcomes at clinic level. Rather than only focussing the intervention on nurses as frontline implementers (and on their clinical supervisors), facility managers were integral to the Project Steering Committee. According to the project champion, their involvement and monitoring role were critical to the successful outcome of the intervention: *‘Facility managers wanted to deliver results – and they knew they were being monitored. People are more responsive when having to report regularly’.*

In addition, targets for improving HIV test uptake amongst STI patients were introduced by top management during the implementation period. Management made this a key performance area against which all facility managers were evaluated. This responsive organisational context is likely to have contributed to normalisation of the PITC intervention.

## Conclusion

This study examined how factors affecting the implementation of a new PITC intervention may have influenced the outcomes of a controlled trial of PITC among STI patients in Cape Town. The study identified a number of facilitators to the normalisation of the new PITC intervention, including strong senior management leadership, implementation support, appropriate accountability mechanisms, an intervention design that was responsive to service needs and congruent with professional practice, positive staff and patient perceptions, and a responsive organisational context. Similar factors (related to characteristics of the intervention, of providers, and of the organisational context) have been identified as important for the successful implementation of new interventions in other studies and also in theoretical frameworks [21,41,42].

The congruence or ‘fit’ of the new intervention with existing clinical practice was an identified key factor in this study, one that has also been identified as important in other NPM studies [23,26]. The study highlighted a tension in achieving this congruence, between promoting ‘interactional workability’ and ‘relational integration’ on the one hand, and achieving ‘skillset workability’ on the other. As discussed earlier, adding more tasks to the clinical guideline may have enhanced interactional workability (by promoting wider acceptability among stakeholders), but lengthening the guideline may have reduced compatibility with existing nurse skillsets and with the structure of the clinical consultation, making efficient deployment more difficult. The continued presence of the ‘counselling’ paradigm within the PITC approach (associated with the requirement for ‘true’ patient informed consent) may also be in tension with the conventional, more directive clinical communication styles of nurses. This presents a challenge that may be difficult for providers to resolve within a standard clinical consultation [43,44], especially if the training does not develop the skills and confidence required to resolve it.

This tension of navigating between provider-centred and patient-centred communication modes has been cited in the HIV literature as a particular challenge in relation to nurse practices in low-and middle income countries (LMICs). A review of studies from sub-Saharan Africa reported that nurses implementing PITC experienced a conflict between the counselling ethos and their perceived professional role as clinical experts and advisors for patients [43]. An ethnographic study of PMTCT nurses and their patients in Tanzania showed the complexity of nurse-patient communication in relation to HIV; in the context of formal ‘counselling’ on infant feeding, nurses were more directive in dispensing information, which patients viewed as the nurse showing concern. Interestingly, these same nurses were found to be more interactive and empathetic in other clinic settings and especially during home visits [44].

Another NPM study in Cape Town (with the same health authority) offers a useful comparison with the finding from this PITC study. The study examined why a patient-centred TB adherence programme failed to be routinised. It identified similar implementation strengths and concluded that the main weakness was an intervention design that was a poor fit with existing nurse perceptions and practice [28]. Similar barriers were identified in a recent Cape Town study investigating the poor coverage and poor fidelity of a new counselling approach to promote patient adherence to antiretroviral treatment [45]. These findings provide support for considering the importance of congruence of intervention design with professional practice, as seen in this PITC study.

Changing practice may be difficult to achieve in an environment where nurses are over-burdened and have low morale, as has been described in many low- and middle-income countries (LMICs), including South Africa [28,46,47]. High workloads and stress levels limit the potential of nurses to respond to the counselling needs of HIV-positive patients, and nurses have called for more training and support to enable them to fulfill this role [43,48]. Based on the challenges observed with deployment of the PITC intervention on the level of the clinical consultation, it is recommended that future PITC interventions and up-scaling pay closer attention, firstly, to the congruence of the new tasks, especially in terms of compatibility with the operational tasks on the level of the clinical consultation, and, secondly, to the need for focussed training to enable efficient deployment of new tasks.

Finally, HIV testing rates for non-pregnant women in South Africa have increased dramatically, from 2.9 million in 2009 to 5.9 million in 2011 [49], largely due to the massive country-wide campaign initiated by the National Department of Health in 2010. Although PITC has been included in the national HIV Testing and Counselling (HCT) policy since 2010, it is unclear to what extent PITC is routinely implemented and what its contribution is to increased HIV testing rates. This will require further monitoring and research.

### Strengths and limitations of this study

This process evaluation used a range of qualitative methods to examine both micro- and macro-level implementation issues, and also drew on the quantitative trial findings. The multi-method approach resulted in a robust and comprehensive evaluation of the intervention. The NPM theoretical framework facilitated a deeper and more dynamic analysis. Use of an evidence-based framework in the first stage analysis mitigated the potential constraints that come with applying one theoretical perspective. The study also highlighted the importance of observation of micro-level clinical practice for generating insights that would have been hard to gain from other sources of information, such as interviews with providers.

The qualitative research was done in parallel to the implementation of the trial, which had the benefit of allowing implementation to be observed in ‘real time,’ but it also reduced the explanatory value of the study as the final trial results were not available during qualitative data collection. This is a dilemma acknowledged by others using process evaluations [5,50].

A challenge with applying the NPM was how to avoid duplication and fragmentation resulting from the overlap between the various constructs and dimensions of the model. For example, congruence of intervention design with professional practice can be analysed under all of the first three constructs. We focussed our analysis of this issue under a single construct, which contributed to ‘skillset workability’ being a disproportionally long discussion. Another limitation was that we were not able to draw in detail upon a comparative analysis of professional and patient accounts for this study, and this will form the basis of a later publication.

To conclude, the use of a theory-driven analysis of implementation processes provides conceptual generalisation that improves the transferability of the results [34]. The research is of relevance to the implementation of HIV testing and to the design and evaluation of complex interventions in other settings.

## Competing interests

The authors declare that they have no competing interests.

## Authors’ contributions

NL, SL and CM conceived of the study and participated in its design. NL was responsible for coordinating the study and data acquisition. NL, SL and CM were responsible for analysis of data, and all authors were involved in data interpretation. NL drafted the main manuscript, and all the authors contributed important intellectual content and revisions. All authors read and approved the final manuscript.
